# A Novel Compliant Connection Mechanism with Thermal Distortion Self-Elimination Function

**DOI:** 10.3390/mi15060774

**Published:** 2024-06-11

**Authors:** Yunyang Huang, Zhanchen Liao, Zhihang Lin, Fahui Feng, Hui Tang

**Affiliations:** 1 State Key Laboratory of Precision Electronic Manufacturing Technology and Equipment, Guangdong University of Technology, Guangzhou 510006, China; 3121000479@mail2.gdut.edu.cn (Y.H.); 3121000168@mail2.gdut.edu.cn (Z.L.); 3120000440@mail2.gdut.edu.cn (F.F.); 2 School of Engineering, University of Warwick, Coventry CV4 7AL, UK

**Keywords:** compliant mechanism, FACT, thermal distortion, OLED inkjet printing

## Abstract

As a novel technology for fabricating large-screen OLED devices, OLED inkjet printing places extreme demands on the positioning accuracy of inkjet printing platforms. However, thermal deformation of the connection mechanism often reduces the printing precision of OLED printing equipment, significantly impacting overall print quality. This study introduces a compliant connection mechanism that achieves precise positioning of the inkjet printing platform and can self-eliminate thermal distortion. The design of the mechanism’s core component is based on the Freedom and Constraint Topology (FACT) principle. This component is constructed from three distinct compliant sections arranged in series, collectively providing three degrees of freedom. Furthermore, the resistance to deformation caused by gravity and other external forces was evaluated by analyzing both vertical and horizontal stiffness. To validate the mechanism’s thermal distortion elimination and gravity resistance capabilities, finite element analysis (FEA) was carried out. The results demonstrate that the mechanism effectively reduces the maximum deformation of the platform by approximately 46% and the average deformation across the entire platform by approximately 59%. These findings confirm that the mechanism has potential in high-precision positioning tasks that need to mitigate thermal distortion.

## 1. Introduction

Advances in display technology have led to a growing demand for display devices, with organic light-emitting diodes (OLEDs) being among the most promising candidates. However, the production of large-scale OLED screens is currently constrained by the high costs associated with fine metal mask (FMM) technology [1]. Inkjet printing technology has emerged as a viable solution to this problem, offering several advantages over FMM technology, such as higher material utilization and the flexibility to produce screens of various sizes [2,3,4,5].

**Figure 1:**
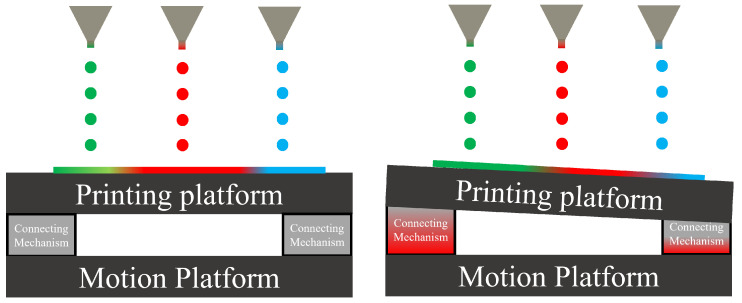
OLED inkjet printing equipment working diagram.

Inkjet printing stands out due to its efficient material utilization and adaptability to various screen sizes. One of the key factors influencing the performance of inkjet printing devices is the precise positioning of liquid droplets. This accuracy heavily depends on the positioning and movement precision of the mechanical displacement platform as shown in Figure 1.

Currently, inkjet printing equipment relies on high-precision air-bearing motion platforms for positioning and movement. However, these platforms have stringent requirements regarding the surrounding gas, ambient temperature, and equipment temperature [6,7]. Consequently, their high cost and strict usage conditions have limited their widespread application in large-scale OLED screen production.

To address these issues, researchers need to propose a cost-effective connection mechanism to replace the air-bearing motion platform, aiming to achieve the stable and precise fixation and positioning of the printing platform while minimizing the need for complex equipment and specialized environmental conditions.

Considering these issues, compliant connection mechanisms have been proposed to align the printing platform and mitigate the adverse effects of temperature rises. Compliant connection mechanisms offer frictionless operation, precision, and durability, making them a common choice in robotics manufacturing and precision instrument applications [8,9,10,11].

Despite significant progress in compliant mechanisms since the pioneering works of Larry L. Howell [8,9], designing such mechanisms remains challenging for engineers. This paper proposes a novel compliant mechanism utilizing theFACT (Freedom and Constraint Topology) method [12,13,14,15,16,17,18,19,20]. Firstly, the design principle of this mechanism is introduced. Then, through mechanical and mathematical modeling, the mechanism’s thermal deformation absorption capabilities are quantified, establishing vertical and horizontal stiffness models to assess the influence of gravity and other forces.Additionally, to validate the proposed model and conclusions, the finite element method is employed. Finally, the results confirm the effectiveness of our stiffness models in meeting the desired standards and demonstrate the mechanism’s remarkable ability to self-compensate for thermal distortions.

## 2. Design Principle of Mechanism

**Figure 2:**
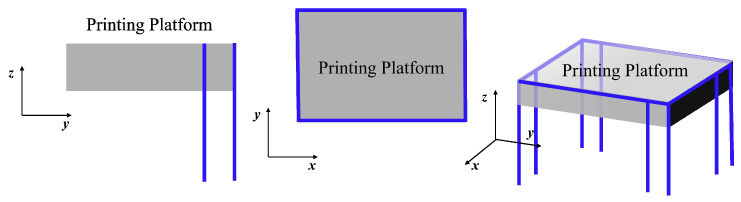
The constraint diagram of the printing platform (the blue line is the constraint line).

Our goal is to create constraints using the principles of exact constraints. These constraints serve to keep the printing platform in a fixed position, preventing any movement between it and the motion platform (Figure 1). Figure 2 illustrates how we will use these constraints to limit the platform’s movement to six degrees of freedom in space. To achieve this, we will designate each constraint on each side of the platform as a separate constraint space. Our ultimate aim is to use this constraint space to design compliant mechanisms.

**Figure 3:**
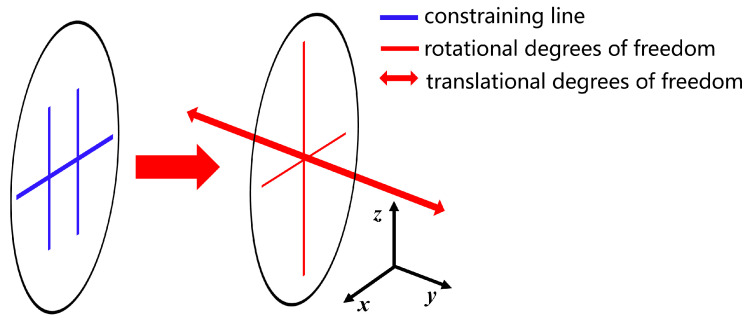
Mapping from constraint space to freedom space.

The FACT method is a useful tool for visualizing the constraint space by mapping it to the degree of freedom (DOF) space. This method is described in several articles, including [12,13,14,15,16,17,18]. The resulting visualization is shown in Figure 3. By applying the principle of FACT, we can deduce that if our mechanism has a freedom space as shown in Figure 3, then it must also have a constraint space as shown in Figure 3 [13].

Notch-type pivots with lumped compliance are commonly used to provide bending and rotation movements, but they have some limitations. For example, they typically have high rotational stiffness, limited stroke length, and high concentrated stress [21,22,23]. These drawbacks restrict their application in high-precision positioning tasks. To overcome these limitations, this study adopts leaf-type pivots with distributed compliance as the primary component of the design.

### 2.1. Design of Compliant Translation Module

**Figure 4:**
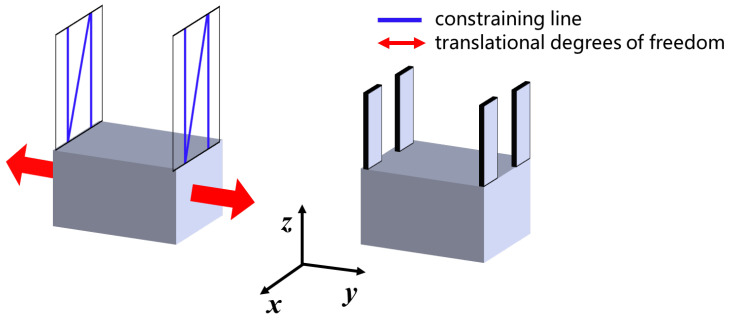
Design flow of compliant translating module.

As described in [13], the compliant translation module aims to enable translational movement while restricting other degrees of freedom. To achieve this, we have designed a constraint space and subsequent compliant translational motion module, which is illustrated in Figure 4.

### 2.2. Design of Compliant Rotary Motion Module

**Figure 5:**
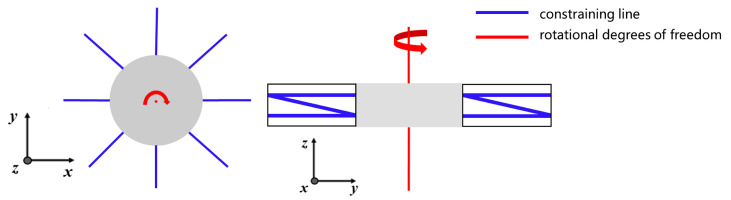
The constraint space design of the compliant rotating module.

To achieve two degrees of freedom in the rotational space, we need two separate compliant rotational motion modules placed in series, each for its respective degree of freedom. The design of the rotational DOF (Domain Of Freedom) module is fundamental to the mechanism’s overall design. Similar to the design of the compliant translation module, we need to create a corresponding constraint space first as shown in Figure 5. The distribution of this constraint space is vital to ensure the mechanism’s efficiency and performance.

To achieve rotational movement around the *X*-axis, a cross-axis flexural pivot can be used. This type of pivot has been extensively studied and modeled in previous research as cited in [11,22,24]. For this particular study, we selected this compliant module to provide rotational freedom around the *X*-axis.

### 2.3. Series Connection and Assembly of Modules

**Figure 6:**
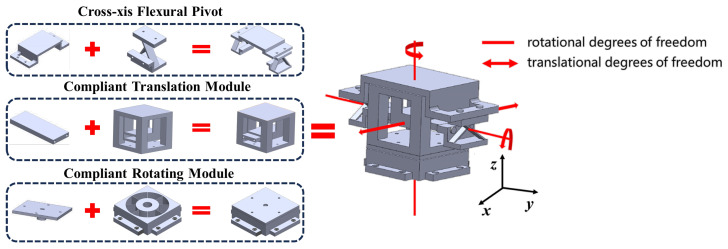
Schematic diagram of assembly relationship between modules and mechanism.

Once all the modules have been designed, it is crucial to inspect the assembly relationship between them. Figure 6 provides a visual representation of this relationship.

## 3. Modeling of Compliant Mechanism

**Figure 7:**
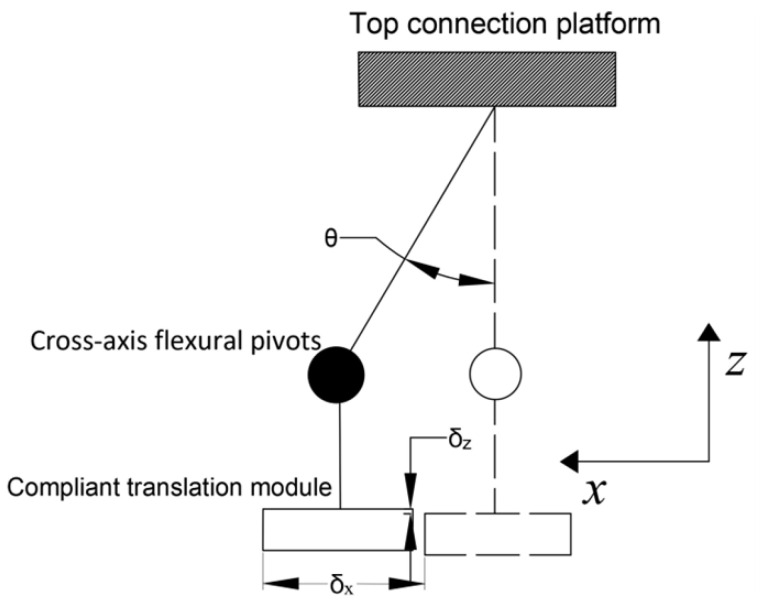
Schematic of deformation of both compliant translation module and cross-axis flexural pivot to absorb thermal stress.

In the context of compliant mechanisms, it is important to consider the principle of self-elimination of thermal distortion. When compliant translation mechanisms or compliant rotation mechanisms are exposed to heat, they will deform accordingly. Due to their significantly lower stiffness in a particular direction compared to other directions, thermal deformation tends to occur in that direction. However, the deformation mode shown in Figure 7 does not negatively affect the accuracy of the positioning or motion of the upper platform. Therefore, this mechanism can be considered a self-elimination mechanism for thermal distortion.

The compliant translational module and the cross-axis flexural pivot’s rotational and translational stiffness are significant parameters to evaluate the mechanism’s capability to dissipate thermal stress. When the motion stiffness of these components decreases, it indicates that the mechanism can better absorb thermal deformation.

### 3.1. Modeling of Compliant Translation Module

**Figure 8:**
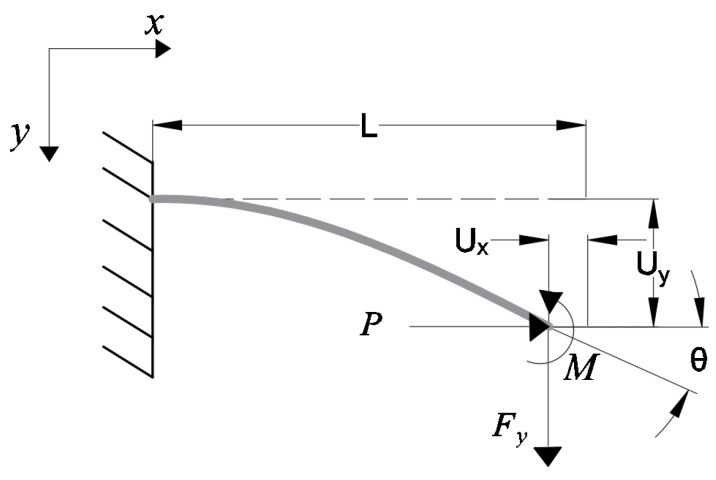
A schematic diagram of a common beam.

Due to the challenges in achieving accurate modeling using the rigid body model (RBM), the beam constraint model (BCM) is often used to complete the modeling of bending-compliant parts [25]. In the compliant translation module, we used BCM to complete the construction. For example, we can examine the stress diagram of an ordinary beam in Figure 8 and obtain the equations for the beam’s displacements and loads as shown in Equation (1):

(1) $$\left[\begin{array}{c} f\\ m \end{array}\right]=\left[\begin{array}{cc} k_{11}^{\left(0\right)} & k_{12}^{\left(0\right)}\\ k_{12}^{\left(0\right)} & k_{22}^{\left(0\right)} \end{array}\right]\left[\begin{array}{c} y\\ \theta \end{array}\right]+P\left[\begin{array}{cc} k_{11}^{\left(1\right)} & k_{12}^{\left(1\right)}\\ k_{12}^{\left(1\right)} & k_{22}^{\left(1\right)} \end{array}\right]\left[\begin{array}{c} y\\ \theta \end{array}\right]$$

where

(2) $$f=\frac{F_{y}L^{2}}{EI_{zz}}\;m=\frac{ML}{EI_{zz}}\;p=\frac{PL^{2}}{EI_{zz}}$$

(3) $$y=\frac{U_{y}}{L}\;x=\frac{U_{x}}{L}$$

**Figure 9:**
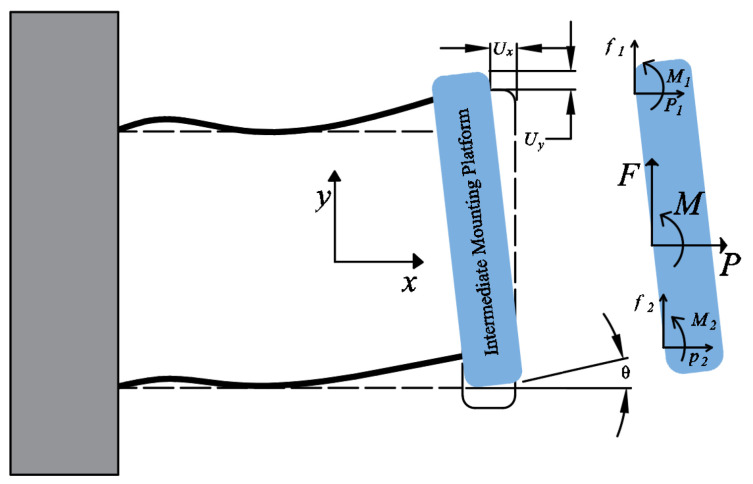
Load and displacement diagram of compliant translation module.

However, the mounting platform in the compliant translation module is subjected to two beam forces on one side. The forces are shown in Figure 9.

Using the deformation geometry of the two beams:

(4) $$x=\frac{\left(x_{1}^{k_{11}^{\left(1\right)}}+x_{2}^{k_{11}^{\left(1\right)}}\right)}{2}+\frac{\left(x_{1}^{g_{12}^{\left(0\right)}}+x_{2}^{g_{12}^{\left(0\right)}}\right)}{2}≜x^{k_{11}^{\left(1\right)}}+x^{g_{12}^{\left(0\right)}}$$

(5) $$\omega \theta =\frac{\left(x_{2}^{k_{11}^{\left(i\right)}}-x_{1}^{k_{11}^{\left(1\right)}}\right)}{2}+\frac{\left(x_{1}^{g_{12}^{\left(0\right)}}+x_{2}^{g_{12}^{\left(0\right)}}\right)}{2}$$

(6) $$y_{1}=y-\frac{\omega \theta^{2}}{2}\;y_{2}=y+\frac{\omega \theta^{2}}{2}⟹y_{1}\approx y_{2}\approx y$$

The characteristic parameters of the beam are in Table 1.

We are using a support plate, so we replace the plate modulus with Young’s modulus. To obtain the load–displacement equation of the platform, we use the equilibrium relation of the force and moment of the middle installation platform. This results in three equilibrium equations, which are connected with the equation of the deformation geometric relation of the beam. Finally, we solve these equations to obtain the load–displacement equation of the platform:

(7) $$\begin{array}{c} \theta =\frac{\left[4{\left(k_{11}^{\left(0\right)}\right)}^{2}+4P\left(k_{11}^{\left(1\right)}k_{11}^{\left(0\right)}\right)+P^{2}{\left(k_{11}^{\left(1\right)}\right)}^{2}+f^{2}g_{11}^{\left(1\right)}d\right]\left[2mk_{11}^{\left(0\right)}-2fk_{12}^{\left(0\right)}+P\left(mk_{11}^{\left(1\right)}-fk_{12}^{\left(1\right)}\right)\right]}{2w^{2}d{\left(2k_{11}^{\left(0\right)}+k_{11}^{\left(1\right)}P\right)}^{3}}=\frac{1}{2w^{2}}\left(t^{2}+y^{2}g_{11}^{\left(1\right)}\right)\left[m-y\left(2k_{11}^{\left(0\right)}+pk_{11}^{\left(1\right)}\right)\right] \end{array}$$

(8) $$\begin{array}{cc} y= & \frac{f-\left(2k_{12}^{\left(0\right)}+pk_{12}^{\left(1\right)}\right)\theta }{\left(2k_{11}^{\left(0\right)}+pk_{11}^{\left(1\right)}\right)}=\frac{f}{\left(2k_{11}^{\left(0\right)}+pk_{11}^{\left(1\right)}\right)}-\frac{\left(2k_{12}^{\left(0\right)}+pk_{12}^{\left(1\right)}\right)}{2w^{2}\left(2k_{11}^{\left(0\right)}+pk_{11}^{\left(1\right)}\right)}\left[t^{2}+\frac{f^{2}g_{11}^{\left(1\right)}}{{\left(2k_{11}^{\left(0\right)}+pk_{11}^{\left(1\right)}\right)}^{2}}\right]\left[m-f\frac{\left(2k_{12}^{\left(0\right)}+pk_{12}^{\left(1\right)}\right)}{\left(2k_{11}^{\left(0\right)}+pk_{11}^{\left(1\right)}\right)}\right]\\ \; & \approx \frac{f}{\left(2k_{11}^{\left(0\right)}+pk_{11}^{\left(1\right)}\right)} \end{array}$$

(9) $$x=\frac{p}{2d}+y^{2}i+\frac{p}{2}y^{2}g_{11}^{\left(1\right)}$$

where

(10) $$p=\frac{{\mathrm{PL}}^{2}}{EI_{zZ}}\;t=\frac{T}{L}\;d=\frac{12}{t^{2}}$$

$E_{Izz}$ represents the moment of inertia for a beam section. To absorb thermal stress, we require low stiffness along the *Y*-axis, and the stiffness *k* is defined as:

(11) $$\frac{1}{k_{f1}}=\frac{y}{f}=\frac{L^{3}}{EI_{ZZ}2k_{11}^{\left(0\right)}+k_{11}^{\left(1\right)}P}$$

### 3.2. Modeling and Analysis of Cross-Axis Flexural Pivot

The analysis of the stiffness of a cross-axis flexural pivot parallels that of a compliant translation mechanism. In this analysis, two supporting beams are modeled as simple beams. The load–displacement relationship at the ends of these beams is determined using Equation (1). Subsequently, the expression for rotational stiffness is derived from the equilibrium relationship of force and moment on the platform connected by these beams as detailed in Equation (12). These steps are elaborated further in [24]:

(12) $$K_{m}=\left[\frac{2\left(9\lambda^{2}-9\lambda +1\right)}{15\cos\alpha }+\lambda \cos\alpha \right]p+8\left(3\lambda^{2}-3\lambda +1\right)$$

To effectively absorb thermal stress and deformation, the compliant rotational motion module must work in tandem with the compliant translational motion module to achieve translational motion. Therefore, analyzing the translational stiffness of the compliant rotational motion module is essential:

(13) $$K_{f2}=\frac{K_{m}}{\lambda \cos\alpha }=\left[\frac{2\left(9\lambda^{2}-9\lambda +1\right)}{15\lambda \cos^{2}\alpha }+1\right]p+\frac{8}{\lambda \cos\alpha }\left(3\lambda^{2}-3\lambda +1\right)$$

**Figure 10:**
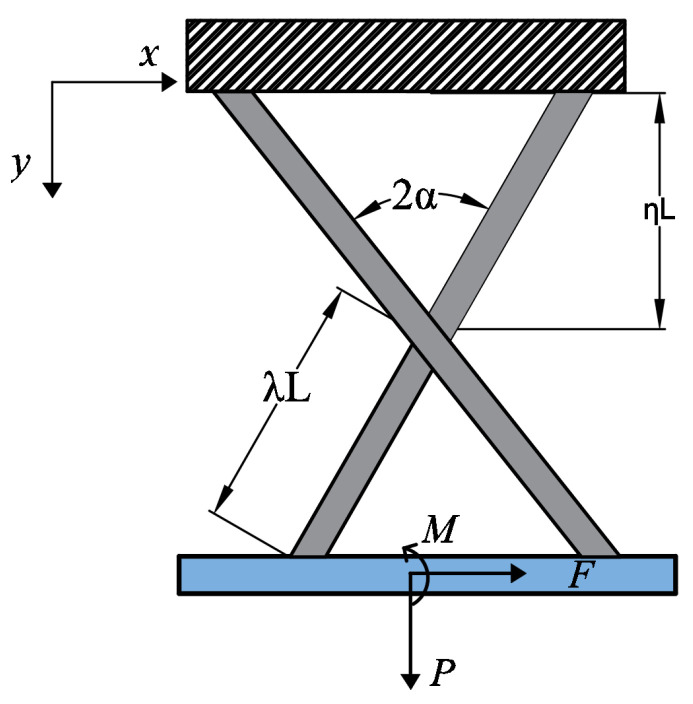
Geometric significance of parameters in cross-axis flexural pivot stiffness analysis.

The geometric parameters in Equation (12) and Equation (13) are shown in Figure 10, respectively.

Since the compliant translational module and the cross-axis flexural pivot are connected in series, the translational stiffness after the combination of the two is

(14) $$K_{1}=\frac{K_{f1}K_{f2}}{K_{f1}+K_{f2}}$$

Measuring the rotational stiffness of the compliant translational module is challenging because the rotational angle θ is linked to both the moment *m* and displacement *y*. In this case, we estimate the rotational stiffness of the two mechanisms by using the rotational stiffness $K_{m}$ of the cross-axis flexural pivot.

### 3.3. Stiffness Modeling in the Vertical Direction

The proposed mechanism has to bear both thermal stress and support the printing platform vertically. We are focusing on examining the stiffness of three compliant mechanisms that are connected in series and are subjected to vertical loads. Based on the beam deflection theory, we have concluded that the cross-axis flexural pivot has a vertical stiffness that remains constant:

(15) $$K_{c}=\frac{\beta \cos^{3}\alpha }{v}$$

where

(16) $$\beta =12{\left(\frac{L}{T_{p}}\right)}^{2}\;v=\frac{\eta }{\lambda }$$

where $T_{p}$ represents the thickness of the beam. In the vertical direction, the two cross-axis flexural pivots are connected in parallel, resulting in a vertical stiffness of $2K_{v}$ for the cross-axis flexural pivot. Furthermore, the cross-axis flexural pivot, the compliant translation module, and the base are connected in series. Therefore, the formula for calculating the vertical stiffness of the entire mechanism is:

(17) $$K_{w}=\frac{1}{\frac{1}{k_{c}}+\frac{1}{k_{m}}+\frac{1}{k_{b}}}$$

The vertical stiffness of the compliant translation module when subjected to only vertical forces is:

(18) $$K_{m}=EA$$

The beams at the base are thick enough in the vertical direction to be considered rigid when subjected to vertical loads from a static perspective. As a result, the vertical stiffness of the final mechanism is:

(19) $$K_{v}=\frac{EA\beta \cos^{3}\alpha }{\frac{1}{2}EAv+\beta \cos^{3}\alpha }$$

### 3.4. Stiffness Modeling in the Horizontal Direction

In Figure 7, we need the mechanism to provide greater stiffness in the Y-direction to ensure that the top of the rotating mechanism does not move when the compliant translation module is heated and translated. We believe that the mechanism can be considered a rigid body in static analysis if its section moment of inertia along the *Y*-axis is large enough. In this case, we only need to consider the stiffness of the base on the *Y*-axis.

When the base is subjected to horizontal force, the deformation of the beam has only two forms—tension and compression. Therefore, we can express the Y-direction stiffness of the base as follows:

(20) $$K_{b}=\sum_{i=1}^{n}EA\left|\cos\theta_{i}\right|$$

where $\cos\theta_{i}$ is the angle between the beam and the *Y* axis, and *n* is the number of beams. Since all beams are parallel to each other, the stiffness of the entire mechanism in the *Y*-axis direction is equal to the sum of the components of all the beams in the *Y*-axis direction. After completing the modeling analysis of the mechanism, we need to determine the optimal design size using the formula obtained from the modeling.

## 4. Determination of Mechanism Size

We need to ensure that the mechanism has the maximum stiffness in the constraint direction and the minimum stiffness in the direction of the degree of freedom so that the mechanism can better absorb the thermal stress. In mathematical terms,

(21) $$\mathrm{Max}\left\{K_{b},K_{v}\right\}$$

(22) $$\mathrm{Min}\left\{K_{1},K_{m}\right\}$$

After understanding some basic optimization objectives and constraints, we determine the geometric parameters that need to be optimized. They are in the cross-axis flexural pivot: λ, *L*, α, and cross-sectional size of the beam: a length *a* and a width *b* (the width *b* needs to be three or more times the length *a*). The compliant translation mechanism can be designed separately from the cross-axis flexural pivot module, so we need to optimize $L_{2}$, $a_{2}$, and $b_{2}$ of the compliant translation module. Due to the large number of parameters to be optimized and the wide range of optimization options, we decided to use the Monte Carlo algorithm to estimate the optimal size model under the constraints. Set the expression of the objective function:

(23) $$\mathrm{Max}K=\omega_{1}K_{b}+\omega_{2}K_{v}-\omega_{3}K_{1}-\omega_{4}K_{m}.$$

where $\omega_{i}\left(i=1,2,3,4\right)$ is the weight. Build the code and model in Matlab (Matlab version 2020b), set the appropriate number of Monte Carlo samples, and then solve it to obtain a maximum value of the objective function. Under the condition of the maximum value of the objective function, the final size of the mechanism is determined as Table 2.

## 5. Simulation Verification

**Figure 11:**
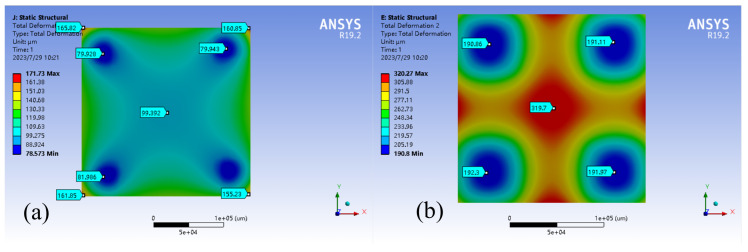
Thermal deformation of the printing platform. (**a**) With the compliant connector; (**b**) Without the compliant connector.

**Figure 12:**
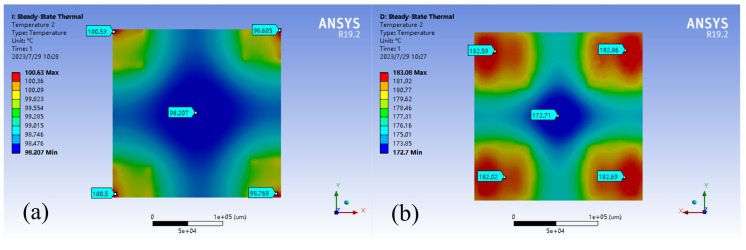
Temperature of the print platform at thermal equilibrium. (**a**) With the compliant connector; (**b**) Without the compliant connector.

**Figure 13:**
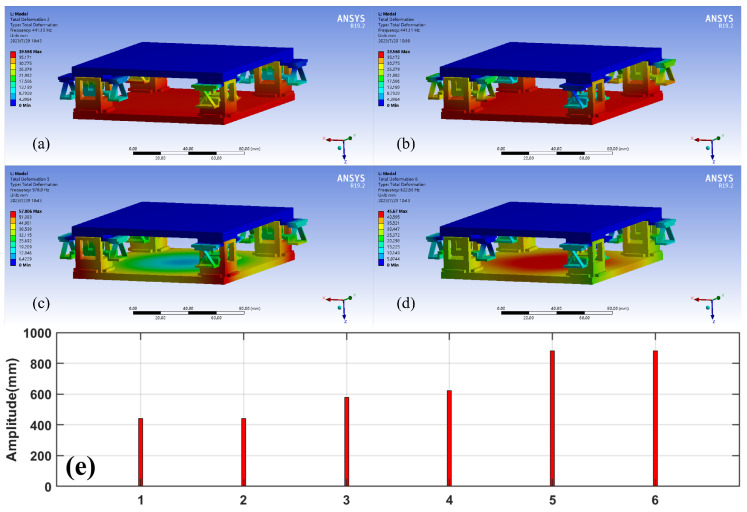
Natural frequencies of the proposed mechanism. (**a**) 1st; (**b**) 2nd; (**c**) 3rd; (**d**) 4th; (**e**) 6 orders.

Steady-state thermal, static structure, and modal analysis was conducted on assemblies using ANSYS 19.2. The temperature of the moving platform at the bottom was set to 200°C to simulate working conditions. The main objective was to evaluate the deformation error of the printing platform caused by thermal deformation of the connecting parts due to temperature increase. The results, shown in Figure 11, Figure 12 and Figure 13, indicate that the traditional rigid connection is superior to the present method.

The results of the finite element analysis conducted at a temperature of 200°C indicate that using a compliant connection module for the printing platform significantly reduced thermal deformation compared to traditional rigid connections. The maximum deformation decreased from 320 μm to 170 μm, a 46% reduction. Additionally, the average deformation decreased from 260 μm to 107 μm, representing a 59% reduction. The deformation shifted from the middle of the sensitive part of the printing platform to the non-sensitive corners. Furthermore, the maximum temperature of the printing platform was reduced from 180°C to 80°C, resulting in a 45% reduction.

The modal analysis revealed that the first two natural frequencies of the mechanism are closely identical (441.11 Hz and 441.13 Hz). This implies that the mechanism exhibits similar vibration characteristics in these two modes, suggesting that their vibrations may interact, leading to more complex vibrations. We discovered that the vibration modes of the printing platform exhibit high stiffness at various vibration frequencies, suggesting that these frequencies have a high degree of rigidity and are resistant to deformation.

## 6. Conclusions

This paper introduces a novel compliant connection mechanism based on the FACT method, aimed at enhancing the performance of OLED inkjet printing devices. The mechanism addresses issues such as misalignment caused by thermal deformation in OLED inkjet printing devices.We developed static and dynamic models for the mechanism, providing force–displacement expressions for the mechanism. With the objectives of maximizing support stiffness and maximizing thermal deformation absorption capacity, we optimized the size of the mechanism using the obtained mathematical model expressions. Finally, we conducted finite element analysis to validate the mechanism. The results indicate that the maximum thermal deformation of the printing platform connected by the compliant connection mechanism is reduced by 46% compared to traditional rigid connections, decreasing from 320 μm to 170 μm. Additionally, the average deformation of the printing platform is reduced by 59%, from 260 μm to 107 μm, compared to traditional rigid connections. This demonstrates the effectiveness of the compliant connection mechanism in reducing thermal distortion. These results meet our design requirements and offer a potential solution for OLED inkjet printing equipment.

## Figures and Tables

**Table 1:** The characteristic parameters of the beam.

Parameter	Value	Parameter	Value
$k_{11}^{\left(0\right)}$	12	$k_{11}^{\left(1\right)}$	1.2
$k_{12}^{\left(0\right)}$	−6	$k_{12}^{\left(1\right)}$	−0.1
$k_{22}^{\left(0\right)}$	4	$k_{22}^{\left(1\right)}$	0.13
$g_{12}^{\left(0\right)}$	0.5		

**Table 2:** Optimal size values of mechanism.

Parameter	Value	Parameter	Value
λ	12	α	0.61
*L*	14.6 mm	*a*	0.8 mm
*b*	3.6 mm	$L_{1}$	7.9 mm
$a_{1}$	0.6 mm	$b_{1}$	2.1 mm

## Data Availability

The original contributions presented in the study are included in the article, further inquiries can be directed to the corresponding authors.
